# Exploring the Link Between Resilience and Disease Severity in Chronic Rhinosinusitis With Nasal Polyps

**DOI:** 10.7759/cureus.78027

**Published:** 2025-01-26

**Authors:** Mihai I Tănase, Mara Tanase, Marcel Cosgarea, Gheorghe Doinel Radeanu, Raluca Maria Hendea, Alma A Maniu

**Affiliations:** 1 Department of Otolaryngology, ”Iuliu Haţieganu” University of Medicine and Pharmacy, Cluj-Napoca, ROU; 2 Department of Anatomical Pathology, “Iuliu Haţieganu” University of Medicine and Pharmacy, Cluj-Napoca, ROU

**Keywords:** cd-risc, crswnp, ki-67 antigen, snot-22 questionnaire, wellness and resilience

## Abstract

Background

Chronic rhinosinusitis with nasal polyps (CRSwNP) significantly affects patients' quality of life, impacting both physical and psychological well-being. This study investigated the potential role of resilience in coping with CRSwNP and its relationship to disease severity and cellular proliferation.

Methodology

Between July 1, 2021, and June 30, 2022, 30 patients diagnosed with CRSwNP were enrolled in a study at Cardiomed Hospital in Cluj-Napoca, Romania. Resilience was assessed using the Connor-Davidson Resilience Scale (CD-RISC), while disease severity was evaluated through patient-reported outcomes (Sino-Nasal Outcome Test (SNOT-22)) and objective measures (Lund-Mackay score). Ki-67 expression was analyzed as a marker of cellular proliferation within nasal polyps.

Results

No significant correlation was observed between resilience and either SNOT-22 scores or Ki-67 expression. However, a strong positive correlation was found between SNOT-22 scores and Lund-Mackay scores, indicating that patients with more severe symptoms also exhibited greater objective disease burden. In addition, a moderate positive correlation was identified between Ki-67 expression and Lund-Mackay scores, suggesting a potential link between cellular proliferation and disease severity.

Conclusion

These findings suggest that resilience may not directly influence symptom severity or cellular proliferation in CRSwNP. Further research with larger sample sizes and longitudinal designs is needed to elucidate the complex interplay between resilience, disease pathophysiology, and patient outcomes in CRSwNP. This will ultimately inform the development of more targeted and effective interventions to improve the quality of life for individuals with this chronic condition.

## Introduction

Chronic rhinosinusitis with nasal polyps (CRSwNP) is a chronic inflammatory condition of the nasal mucosa and paranasal sinuses characterized by the presence of nasal polyps, which are abnormal growths of tissue that can obstruct the nasal passages and sinuses. The symptoms of CRSwNP can vary depending on the severity of the condition but may include nasal congestion, runny nose, facial pain or pressure, loss of smell, and headaches. The exact cause of CRSwNP is unknown, but it is thought to be related to a combination of genetic, environmental, and immunological factors [1].

The impact of CRSwNP extends beyond physical symptoms, significantly affecting patients' emotional well-being and overall quality of life. Studies have shown that individuals with CRSwNP often experience higher rates of anxiety, depression, and sleep disturbances, leading to a reduced quality of life compared to the general population which underscores the importance of considering the psychological impact of CRSwNP and exploring potential interventions that can improve patients emotional and mental health [2].

Resilience, a multifaceted construct encompassing psychological, emotional, and biological factors, plays a crucial role in an individual's ability to cope with stress, adversity, and chronic health conditions. In the context of CRSwNP, resilience may be particularly important due to the significant impact of the disease on physical and emotional well-being. It is plausible that individuals with higher resilience may experience a lower symptom burden or exhibit a different inflammatory profile [3].

Beyond the individual burden, CRSwNP poses a substantial economic burden on healthcare systems and society. The direct costs associated with CRSwNP, including diagnosis, treatment (medications, surgeries), and ongoing management, are considerable. Furthermore, indirect costs arise from lost productivity due to work absenteeism and presenteeism (reduced work efficiency due to symptoms). The economic impact of CRSwNP emphasizes the need for effective strategies to prevent, manage, and potentially even cure this chronic condition [4].

In the context of CRSwNP, the pursuit of accurate diagnoses and effective treatments is crucial for improving patient outcomes and quality of life. By improving diagnostic capabilities, medical professionals can identify CRSwNP with greater precision, enabling prompt and targeted treatment. This can lead to better patient outcomes, as interventions can be tailored to individual needs. Additionally, improved training equips healthcare providers with the knowledge and skills to make informed decisions, interpret diagnostic results accurately, and deliver high-quality care. The importance of better diagnosis and training lies in their potential to empower healthcare systems to provide more effective and efficient care, ultimately improving patient outcomes and overall quality of life [5].

This study aimed to explore the relationship between resilience as an individual’s ability to cope with stress and adversity, disease severity, and cellular proliferation in patients with CRSwNP. We examined the correlations between resilience scores (CD-RISC), patient-reported symptom severity (Sino-Nasal Outcome Test (SNOT-22)), objective disease severity (Lund-Mackay score), and the percentage of Ki-67 positive cells in nasal polyp tissue [6].

## Materials and methods

This study was a prospective analysis of 30 patients diagnosed with CRSwNP. The inclusion criteria were patients with ages between 18 and 65 years, with a minimum duration of symptoms of at least 6 months, with the presence of bilateral multiple nasal polyps. The exclusion criteria were unwillingness to participate, patients with other nasal conditions, pregnant or breastfeeding patients, and patients with cognitive impairment or psychiatric disorders. All patients underwent a comprehensive clinical evaluation, including clinical examination, nasal endoscopy, and computer tomography (CT) scan. This study was approved by the Ethics Committee of “Iuliu Hațieganu” University of Medicine and Pharmacy, number 255 from 30.06.2021. All patients provided written informed consent for the use of their biopsical fragments, which were obtained during their already scheduled surgeries for CRSwNP. No additional biopsies were performed for the purpose of this study. 

SNOT-22 questionnaire

The SNOT-22 questionnaire is a validated instrument used to assess the severity of symptoms in patients with CRSwNP. It consists of 22 questions related to nasal symptoms, such as congestion, runny nose, facial pain, and loss of smell. Each question is scored on a scale of 0 to 5, with higher scores indicating more severe symptoms. The total SNOT-22 score is calculated by summing the scores for all 22 questions, resulting in a range from 0 to 110. 

Connor-Davidson Resilience (CD-RISC) scale

The CD-RISC is a widely used self-report measure of resilience. It consists of 25 items that assess an individual's ability to cope with stress and adversity. Each item is rated on a five-point Likert scale, ranging from 0 (not true at all) to 4 (true nearly all the time). The total CD-RISC score is calculated by summing the scores for all 25 items, resulting in a range from 0 to 100, with higher scores indicating greater resilience [7].

Lund-Mackay score

The Lund-Mackay score is a radiographic scoring system used to evaluate the extent of sinus opacification on CT scans. It assigns points to each sinus (maxillary, ethmoid, frontal, and sphenoid) based on the degree of opacification, ranging from 0 (no opacification) to 2 (complete opacification). The total Lund-Mackay score is calculated by summing the scores for all sinuses, resulting in a range from 0 to 24 [8]. 

Ki-67 expression

Ki-67 is a nuclear protein that is strictly associated with cell proliferation and is expressed throughout the active phases of the cell cycle (G1, S, G2, and mitosis). A semi-quantitative method was employed to assess Ki-67 expression. This involved counting Ki-67 positive cells in 1000 cells observed at 400x magnification following immunohistochemical staining with a Ki-67 monoclonal antibody (clone SP6, Vitro S.A., Spain; lot number 03100066S, expiration date 30.04.2023) and expressing it as a percentage of total cells.

Statistical analysis

Statistical analysis was performed using IBM SPSS Statistics for Windows, Version 25.0 (released 2017, IBM Corp., Armonk, NY). Spearman's rank correlation coefficient was used to assess the correlations between CD-RISC scores, SNOT-22 scores, Lund-Mackay scores, and Ki-67 (%). A p-value of less than 0.05 was considered statistically significant. 

## Results

The study population consisted of 30 patients diagnosed with CRSwNP. The mean age of the participants was 45.2 years (SD = 12.5), with 65% being female. The mean SNOT-22 score was 48.67 (SD = 7.81), the mean CD-RISC score was 73.27 (SD = 7.93), and the mean Ki-67 (%) was 18.33 (SD = 6.96).

Out of the 30 patients selected for the study, 12 were smokers, defined as regular users of tobacco products; 18 non-smokers; six chronic alcohol users, defined as consuming over the recommended limit of seven drinks per week for women and 14 drinks per week for men; 24 non-drinkers; 10 patients diagnosed with asthma; and 11 non-steroidal anti-inflammatory drug (NSAID) intolerant.

The average SNOT-22 score was 48.46, the average CD-RISC score was 73.03, and the average Ki-67 (%) was 18.06.

Spearman's rank correlation analysis was used to examine the relationships between resilience (CD-RISC score), patient-reported symptom severity (SNOT-22 score), objective disease severity as assessed by the Lund-Mackay score, and the Ki-67 proliferation index in nasal polyp tissue.

**Figure 1:**
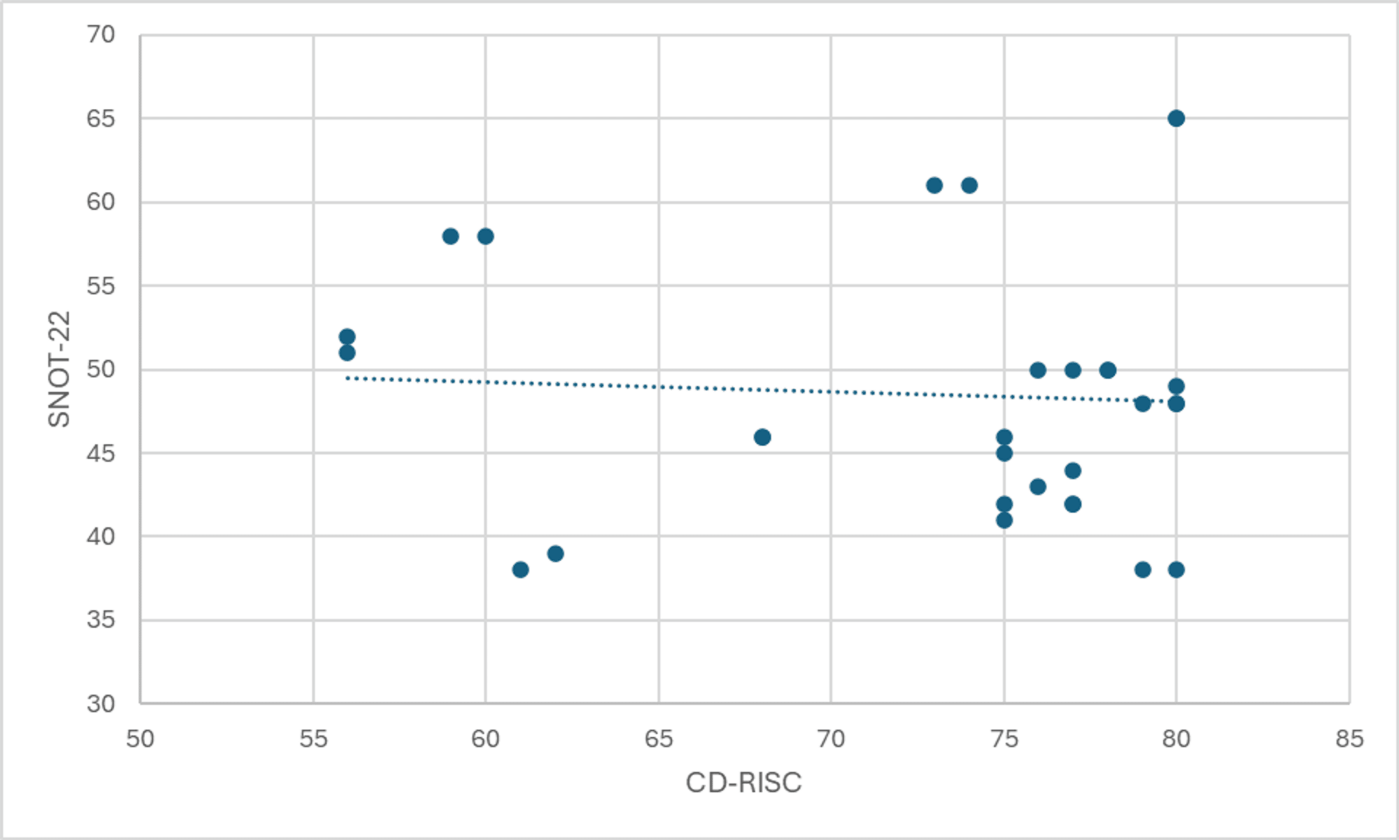
Correlation between the Sino-Nasal Outcome Test (SNOT-22) scores and Connor-Davidson Resilience Scale (CD-RISC) scores

**Figure 2:**
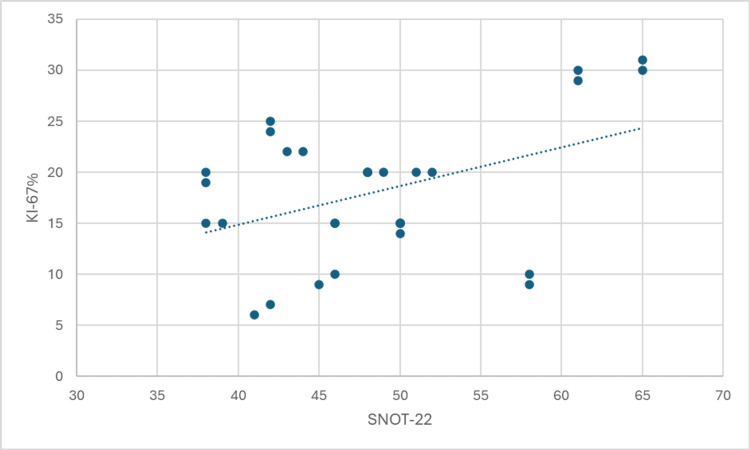
Correlation between Ki-67 (%) and Sino-Nasal Outcome Test (SNOT-22) scores

**Figure 3:**
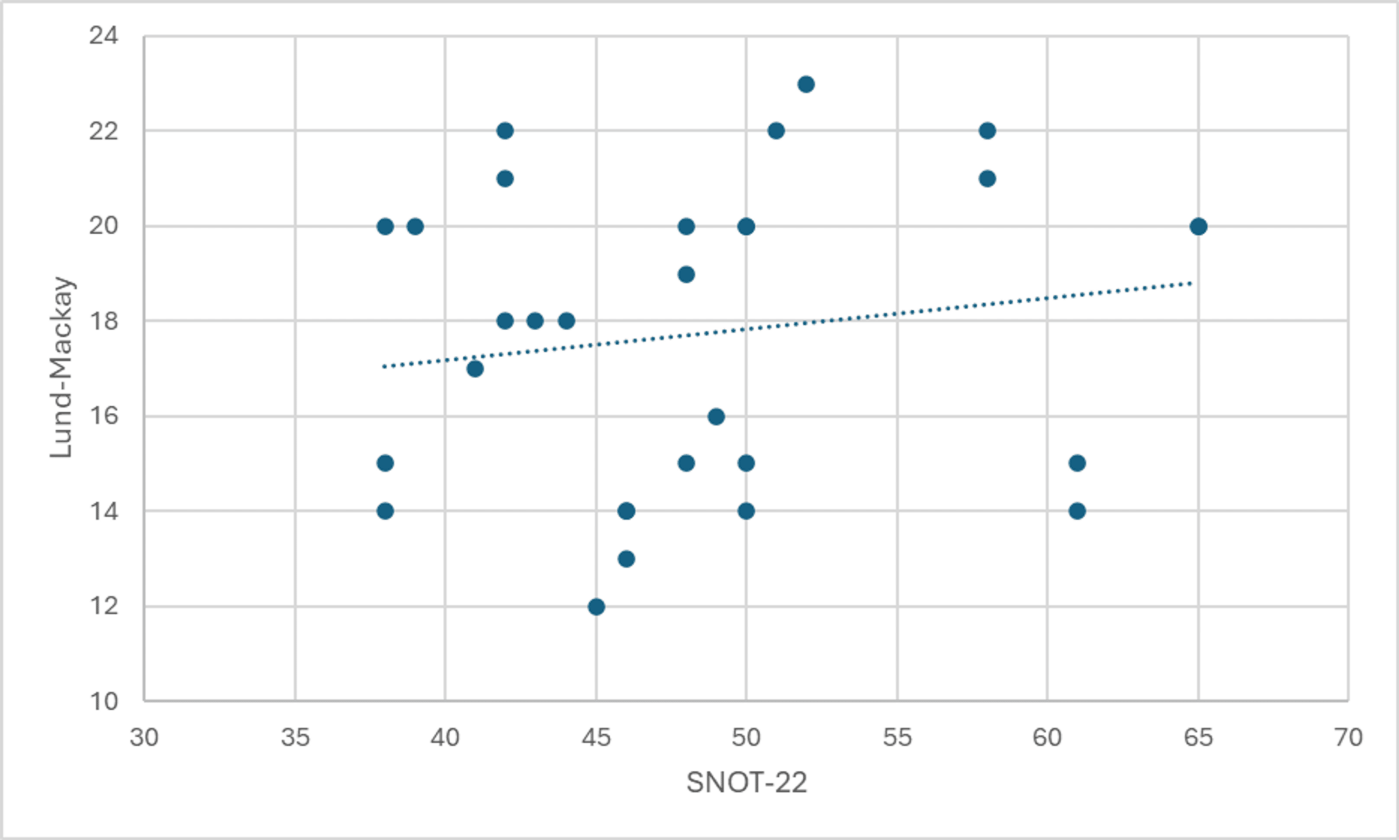
Correlation between Lund-Mackay scores and Sino-Nasal Outcome Test (SNOT-22)

**Figure 4:**
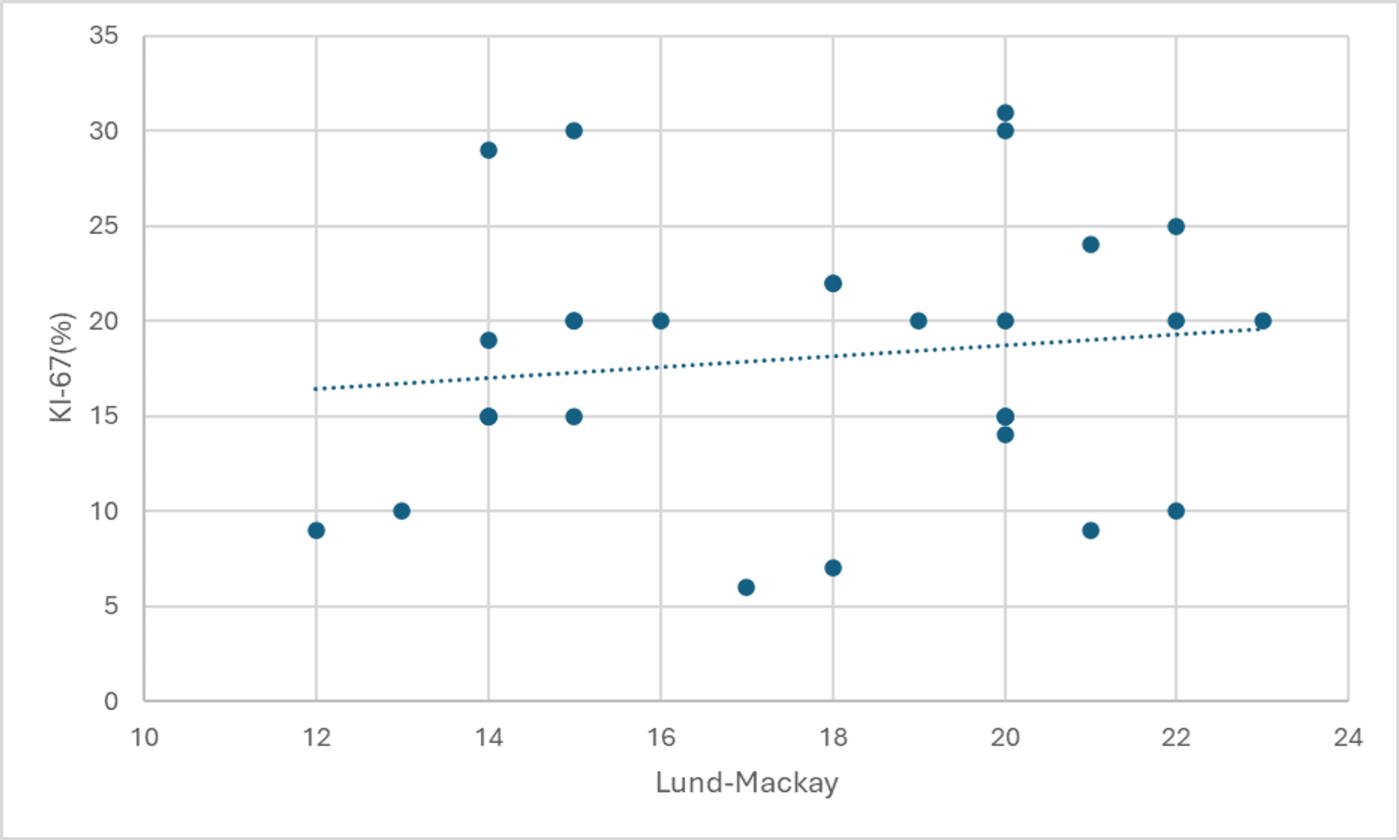
Correlation between Ki-67 (%) and Lund-Mackay scores

The results of the Spearman's rank correlation analysis are presented in Figures 1-4. These figures illustrate the strength and direction of the relationships between the variables of interest. Each figure represents a specific correlation analysis, with Spearman's rho coefficient and the associated p-value provided to indicate the strength and statistical significance of the relationship. 

The analysis revealed a negligible correlation between resilience (CD-RISC score) and patient-reported symptom severity (SNOT-22 score), as indicated by a Spearman's rho of -0.054 (p = 0.778). This suggests that there is no significant relationship between an individual's level of resilience and the severity of their symptoms. 

A weak positive correlation was observed between Ki-67 percentage and SNOT-22 scores (Spearman's rho = 0.268, p = 0.152), indicating that there may be a slight tendency for higher Ki-67 expression to be associated with more severe symptoms, but this relationship was not statistically significant. 

A moderate, statistically significant positive correlation was found between SNOT-22 scores and Lund-Mackay scores (Spearman's rho = 0.565, p = 0.001). This indicates that patients with higher symptom severity tend to have more extensive disease on CT scans. 

A weak, non-significant positive correlation was observed between Ki-67 expression and Lund-Mackay scores (Spearman's rho = 0.122, p = 0.518), suggesting a possible link between cellular proliferation and disease extent, but this requires further investigation. 

## Discussion

This study investigated the potential interplay among psychological resilience, disease severity, and cellular proliferation in patients with CRSwNP. Specifically, we examined the relationship between resilience scores and various indicators of disease activity, including patient-reported symptom severity, objective measures of disease burden, and the Ki-67 proliferation index in nasal polyp tissue. This investigation aimed to elucidate the role of resilience in the context of CRSwNP and its potential influence on disease progression and patient outcomes [9].

The analysis revealed no statistically significant association between resilience scores and either patient-reported symptom severity (SNOT-22) or the Ki-67 proliferation index in nasal polyp tissue. This suggests that psychological resilience may not exert a direct, independent influence on these specific aspects of CRSwNP [10].

Interestingly, a strong positive correlation was observed between SNOT-22 scores and Lund-Mackay scores, indicating concordance between subjective symptom experience and objective radiographic findings. This finding is consistent with previous research demonstrating a relationship between patient-reported outcomes and the extent of disease visualized on CT scans. This underscores the validity of both subjective and objective measures in assessing disease burden in CRSwNP [11].

A moderate positive correlation was observed between Ki-67 expression in nasal polyp tissue and Lund-Mackay scores. This finding suggests a potential association between cellular proliferation and the radiographic extent of disease in CRSwNP. Further research is needed to elucidate the nature of this relationship and to determine whether Ki-67 expression could serve as a prognostic biomarker for disease severity or treatment response [12].

Ki-67 is a well-established marker of cellular proliferation. Its expression in nasal polyp tissue can provide insights into the underlying inflammatory processes and disease activity in CRSwNP [13]. Previous studies have demonstrated variability in Ki-67 expression among CRSwNP patients, with higher expression potentially indicating increased disease severity and a higher risk of recurrence following treatment. However, the complex interplay between Ki-67 expression, resilience, and other clinical factors remains to be fully elucidated [14].

Given the complex interplay between psychological and biological factors in CRSwNP, it is essential to elucidate the relationships between resilience, symptom burden, disease severity, and cellular proliferation [15]. This study sought to explore these relationships in a cohort of CRSwNP patients to identify potential areas for intervention and improve patient care. By examining these interconnected factors, we aimed to contribute to a more comprehensive understanding of CRSwNP and its impact on patients' overall well-being [16].

There is a need for more precise diagnostic and therapeutic approaches in CRSwNP. Current diagnostic tools, while helpful, may not fully capture the complex interplay of inflammation, immune responses, and patient-specific factors contributing to disease development and progression [17]. Advancements in diagnostic techniques, such as biomarkers or imaging modalities that can accurately assess the underlying inflammatory processes, could lead to more targeted and effective treatments. In addition, the development of novel therapies that address the complex pathophysiology of CRSwNP, including the role of psychological factors like resilience, is crucial for improving patient outcomes and reducing the burden of this chronic condition [18].

This study contributes to the growing body of knowledge regarding the complex interplay of factors influencing CRSwNP. While our findings suggest that resilience may not directly translate to reduced symptom severity or lower Ki-67 expression, the intricate connections between psychological and biological aspects of CRSwNP underscore the need for a holistic approach to patient management [19].

It is important to acknowledge the limitations of this study. First, the sample size was relatively small, which may limit the generalizability of the findings. Future studies with larger and more diverse cohorts are needed to confirm these results. Second, the study design was cross-sectional, which cannot establish causality between resilience and disease outcomes. Longitudinal studies are necessary to investigate the temporal relationship between resilience and CRSwNP progression. Third, resilience was assessed using a self-report measure, which may be subject to response bias. Future studies could incorporate multiple measures of resilience, including behavioral and physiological indicators, to provide a more comprehensive assessment. Finally, this study focused on a specific population of patients with CRSwNP, and the findings may not be applicable to other chronic conditions. Further research is needed to examine the role of resilience in other patient populations [20].

## Conclusions

This preliminary investigation explored the intricate interplay among psychological resilience, disease severity, and cellular proliferation in patients with CRSwNP. While no significant direct relationship between resilience and either subjective symptom burden or Ki-67 expression was observed, the study was limited by a small sample size. This suggests that inherent psychological resilience may not substantially influence these specific aspects of CRSwNP, underscoring the need for further confirmatory research with larger patient populations. Despite these limitations, this research provides valuable insights into the complex dynamics of resilience, disease severity, and cellular activity in CRSwNP. Future research should prioritize larger-scale studies, longitudinal data collection, and interventions designed to enhance psychological resilience to elucidate the role of psychological factors in CRSwNP. This will help inform the development of more holistic, patient-centered approaches to care, and could lead to interventions that not only target the physical manifestations of CRSwNP but also bolster patients' psychological well-being and resilience, thereby improving their overall quality of life.
